# Supplementary material for: A protein sequence-based deep transfer learning framework for identifying human proteome-wide deubiquitinase-substrate interactions
Source: Nat Commun. 2024 May 28;15:4519. doi: 10.1038/s41467-024-48446-3 (PMC11133436; doi:10.1038/s41467-024-48446-3)
Supplement: Supplementary file 1 — Supplementary Information [file 41467_2024_48446_MOESM1_ESM.pdf]

Supplementary Information

A protein sequence-based deep transfer learning framework for identifying human proteome-wide deubiquitinase-substrate interactions

Yuan Liu[1], Dianke Li[1,2], Xin Zhang[1], Simin Xia[1,3], Yingjie Qu[1], Xinping Ling[1,4], Yang Li[1], Xiangren Kong[1], Lingqiang Zhang[1], Chun-Ping Cui[1*], and Dong Li[1*]

[1] State Key Laboratory of Medical Proteomics, Beijing Proteome Research Center, National Center for Protein Sciences (Beijing), Beijing Institute of Lifeomics, Beijing 102206, China;

[2] State Key Laboratory of Farm Animal Biotech Breeding, College of Biological Sciences, China Agricultural University, Beijing 100193, China;

[3] School of Basic Medical Sciences, Anhui Medical University, Hefei 230032, China;

[4] College of Life Sciences, Hebei University, Baoding 071002, China;

Yuan Liu, Dianke Li and Xin Zhang contributed equally to this work.

* To whom correspondence should be addressed.

Dong Li, E-mail: lidong.bprc@foxmail.com.

Chun-Ping Cui, E-mail: cui_chunping2000@aliyun.com.

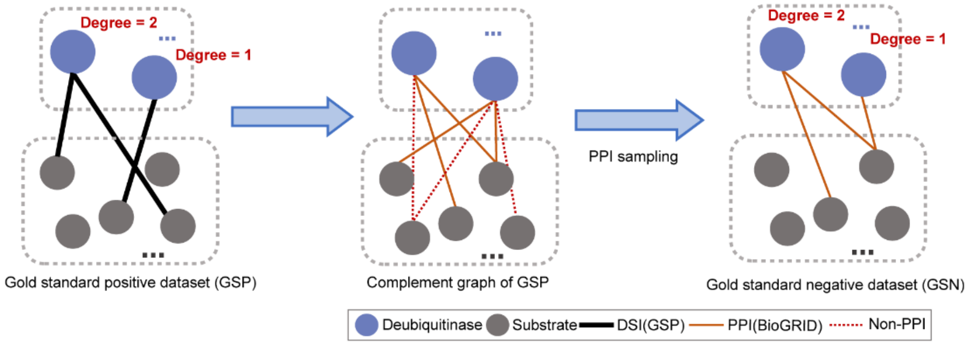

Supplementary Fig.1: Construction of the gold standard negative dataset.

We constructed the negative set by randomly sampling an equal number of nodes from the complement graph of the known DSI network, ensuring identical DUB connectivity distributions between the negative and positive sets. All interactions within the negative set were derived from PPI data in the BioGRID database (***Nucleic Acids Res.*** 2006, 34: D535).

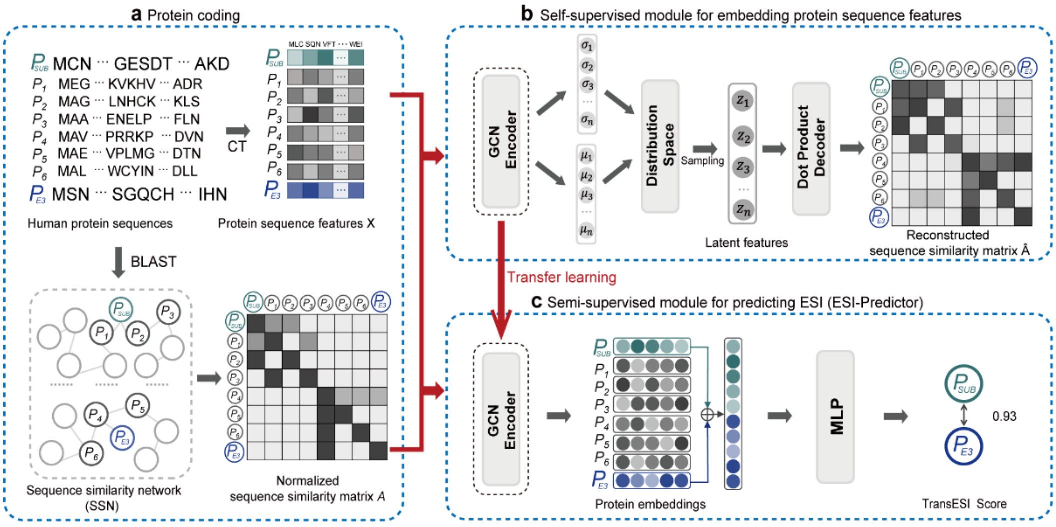

Supplementary Fig.2: Framework of TransESI model. TransESI has the same framework as TransDSI. The model comprises of three components: (**a**) Protein coding: This module takes the primary structures of human proteins including E3s (P_{E3}), substrates (P_{SUB}) and other proteins (P_1-P_6) as input and generates a variety of features for use in downstream deep learning modules. The amino acid sequences of proteins are encoded using the CT method and serve as protein sequence features X (darker colors indicate higher conjoint triad frequency). Additionally, an SSN was created using BLAST and transformed into a normalized sequence similarity matrix A (darker colors indicate higher similarity). (**b**) Self-supervised module for embedding protein sequence features: This module employs a VGAE consisting of a GCN encoder and a dot product decoder, which is used to generate a pre-trained encoder based on the evolutionary information from the SSN and the protein sequence features (see Methods for details of σ, μ , z and $\hat{A}$). (**c**) Semi-supervised module for predicting ESI (ESI-Predictor): The GCN encoder of ESI-Predictor is initialized using the parameters transferred from the self-supervised module to produce the protein embeddings. The embeddings of E3s and their corresponding substrates are concatenated and utilized for fine-tuning the semi-supervised module. The final prediction score ("TransESI Score") for each candidate ESI is obtained by feeding the concatenated embeddings to an MLP.

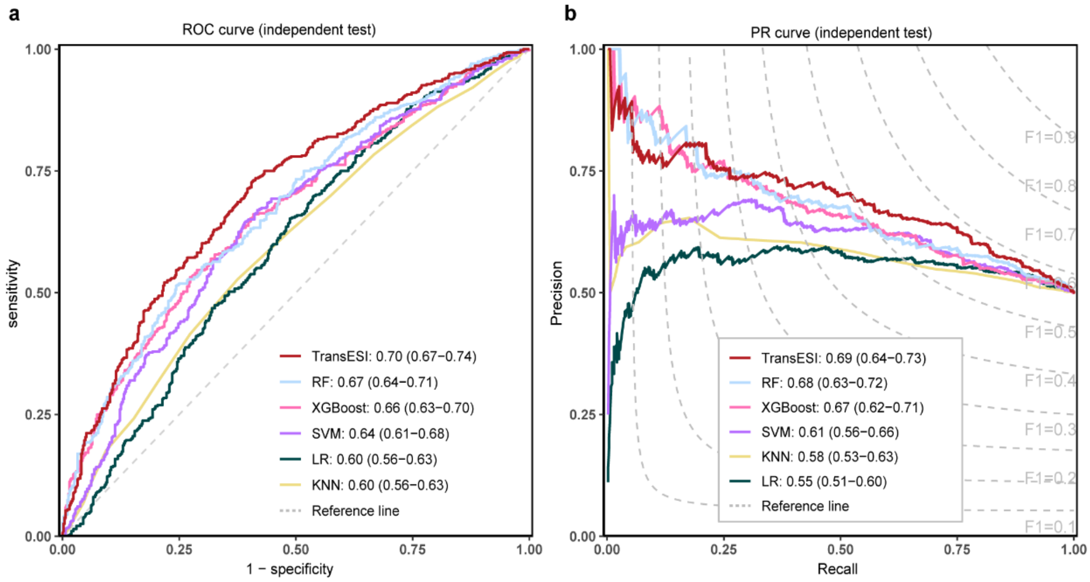

Supplementary Fig.3: Evaluation of the performance of TransESI. We constructed and assessed TransESI models following the same protocol as TransDSI. TransDSI deep learning framework demonstrates satisfactory performance on ESI prediction with the TransESI model achieving an AUROC of 0.70, surpassing other machine learning methods in predictive efficiency. (**a, b**) Performance of various models (TransESI and five machine learning models) for the prediction of ESIs, evaluated through independent test. Five machine learning models are random forest (RF), support vector machine (SVM), eXtreme gradient boosting (XGBoost), logistic regression (LR), and K-nearest neighbors (KNN). The ROC curves of the assessment models demonstrate sensitivity and specificity (**a**) and the PR curves of the assessment models precision and recall (**b**) against a particular prediction score cutoff, with each point on the curves representing the respective values. The 95% confidence intervals (95% CIs) of the sensitivity (precision) at the given specificity (recall) points are computed. The reference line indicates a non-informative prediction with an AUROC of 0.5 (**a**) or a prediction with a constant F1 score across different thresholds (**b**). Source data are provided as a Source Data file.

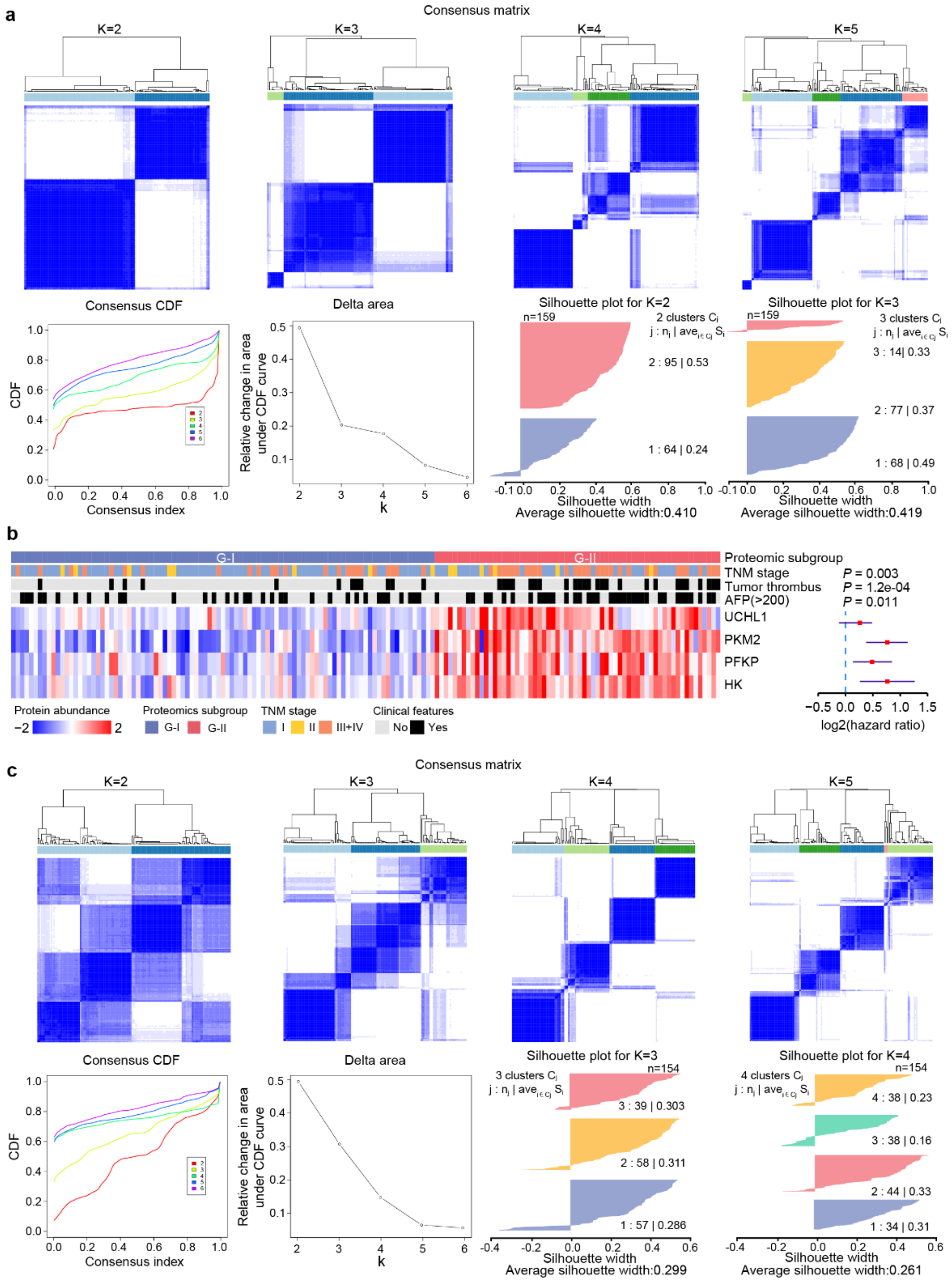

Supplementary Fig.4: Consensus clustering for proteomics data in CHCC-HBV cohort, related to Fig. 5 and 6.

(**a**) Results of consensus clustering applied to proteomics data from the CHCC-HBV cohort (n = 159). The subgroups are identified based on the abundance of UCHL1 and PKM2 using K-means consensus clustering (Methods). The optimal value of k is determined through testing values from 2 to 5 and consensus clustering is performed using 1,000 resampled datasets. The figure includes

consensus matrices, consensus cumulative distribution function (CDF) plots, delta area plots, and silhouette plots for k = 2 and 3.

(**b**) We presented a heatmap displaying the key signaling cascade proteins in the glycolytic pathway, with columns representing samples and rows representing proteins. The middle panel of the heatmap annotates the associations of proteomic subtypes with clinicopathologic factors (Kruskal-Wallis test).

(**c**) Results of consensus clustering applied to proteomics data from the CHCC-HBV cohort (n = 154). The subgroups are identified based on the abundance of USP22 and AR using PAM consensus clustering (Methods). The optimal value of k is determined through testing values from 2 to 5 and consensus clustering is performed using 1,000 resampled datasets. The figure includes consensus matrices, consensus cumulative distribution function (CDF) plots, delta area plots, and silhouette plots for k = 3 and 4. Source data are provided as a Source Data file.

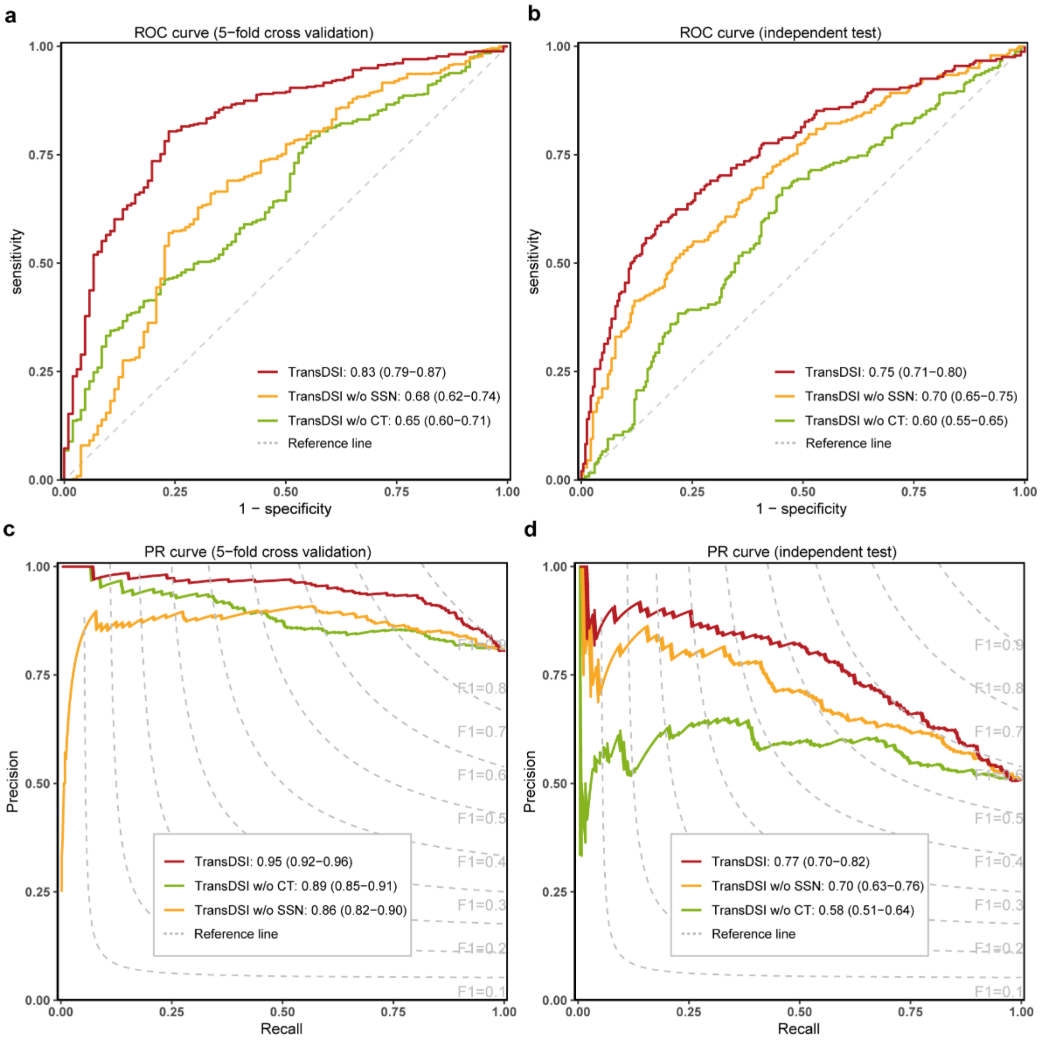

Supplementary Fig.5: Feature importance analysis for TransDSI.

To independently evaluate the impact of both features used by TransDSI (CT-encoded protein feature vectors and SSN based on sequence similarity), we constructed two additional models: 1) TransDSI w/o SSN model: Utilizes CT-encoded sequence features while excluding SSN network information; 2) TransDSI w/o CT model: Employs SSN features while eliminating CT-encoded data. Employing 5-fold cross-validation and independent test, we found that removing either the SSN or CT-encoded sequence information significantly reduced the model's predictive performance. TransDSI, which encompasses both features, demonstrated the highest prediction accuracy among the three models.

(**a-d**) Performance of three models (TransDSI, TransDSI w/o SSN, TransDSI w/o CT) for the prediction of DSIs, evaluated through 5-fold cross-validation and independent test, respectively. The ROC curves of the assessment models demonstrate sensitivity and specificity (**a, b**) and the PR curves of the assessment models precision and recall (**c, d**) against a particular prediction score cutoff, with

each point on the curves representing the respective values. The 95% confidence intervals (95% CIs) of the sensitivity (precision) at the given specificity (recall) points are computed. The reference line indicates a non-informative prediction with an AUROC of 0.5 (**a, b**) or a prediction with a constant F1 score across different thresholds (**c, d**). CT, conjoint triad; SSN, sequence similarity network. Source data are provided as a Source Data file.

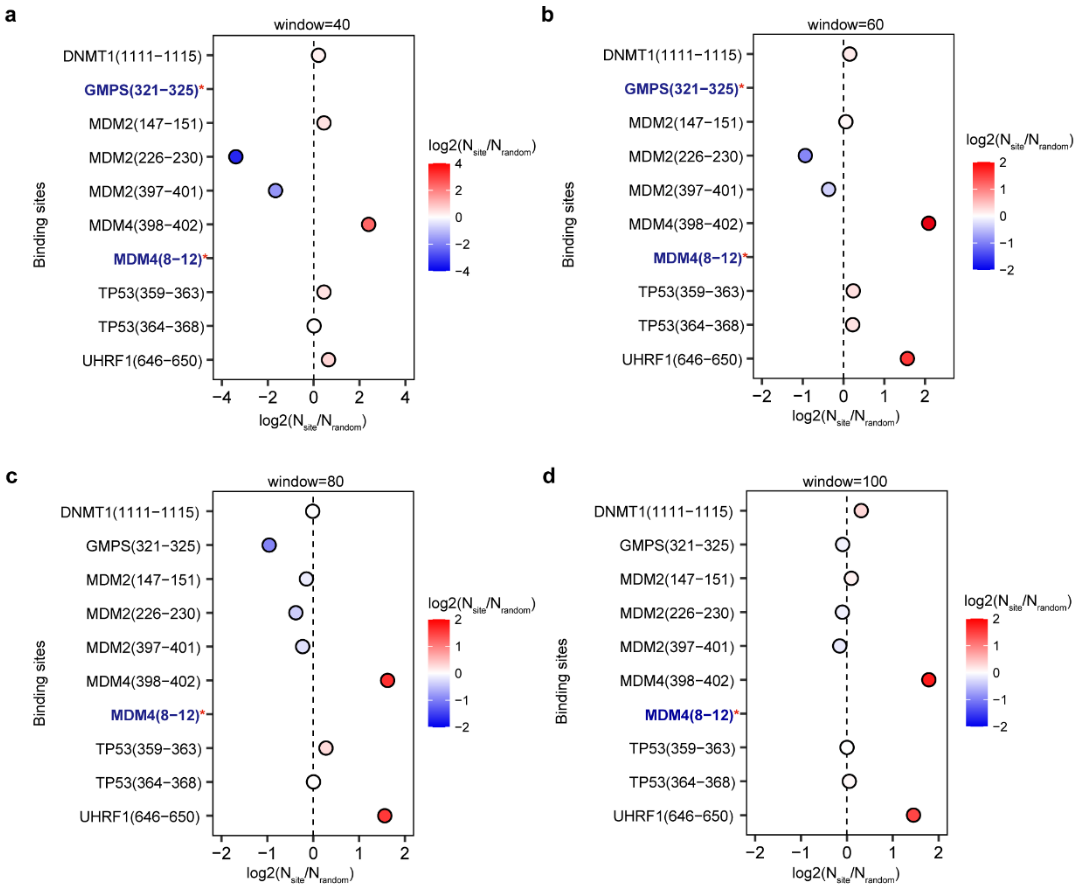

Supplementary Fig. 6: Enrichment analysis of ubiquitination sites within all known DSI binding regions.

All nine DSI binding regions were compiled from literatures, including one DUB (USP7) and its five substrates DNMT1/UHRF1/MDM2/MDM4/TP53(**Supplementary Data 7**). We investigated the regions of 40/60/80/100 amino acid residues centered on the DUB-substrate binding region. We found that the number of ubiquitination sites in this region did not show a significant enrichment or depletion trend compared to those in randomly selected regions from the same substrate protein. Scatter plots depict the log2 ratio of the number of ubiquitination sites around known DUB substrate binding regions versus random regions, across varying window sizes (**a**: 40, **b**: 60, **c**: 80, **d**: 100). (Red: more ubiquitination sites around binding region than random; blue: fewer ubiquitination sites around binding region than random). N_{site}: number of ubiquitination sites around known substrate binding region; N_{random}: number of ubiquitination sites around random region. Gray dashed reference line: $N_{site} = N_{random}$. Red asterisk: no ubiquitination sites in the binding region (N_{site}=0). Source data are provided as a Source Data file.
