# Supplementary material for: A protein sequence-based deep transfer learning framework for identifying human proteome-wide deubiquitinase-substrate interactions
Source: Nat Commun. 2024 May 28;15:4519. doi: 10.1038/s41467-024-48446-3 (PMC11133436; doi:10.1038/s41467-024-48446-3)
Supplement: Supplementary file 3 — Description of Additional Supplementary Files [file 41467_2024_48446_MOESM3_ESM.pdf]

Description of Additional Supplementary Files:

Supplementary Data 1: Gold standard positive dataset.

Supplementary Data 2: The comparison of the performance of TransDSI, UbiBrowser, and various machine learning methods.

Supplementary Data 3: The performance evaluation across diverse negative/positive ratios.

Supplementary Data 4: Predicted DUB-substrate interaction dataset (PDSID).

Supplementary Data 5: Gene Ontology (GO) term enrichment analysis for deubiquitinases (DUBs), known DUB substrates, and predicted DUB substrates.

Supplementary Data 6: Contributing residues for DUBs and their corresponding substrates involved in Gold standard positive dataset.

Supplementary Data 7: Experimentally validated DSI binding sites reported in the literature.

Supplementary Data 8: Relationship between amino acids and CT classes.
